# Effect of somatic antigens of *Dirofilaria repens* adult worms on angiogenesis, cell proliferation and migration and pseudo-capillary formation in human endothelial cells

**DOI:** 10.1186/s13071-023-05726-z

**Published:** 2023-03-16

**Authors:** María del Pilar Pérez Rodríguez, Claudia Alarcón-Torrecillas, Miguel Pericacho, Iván Rodríguez-Escolar, Elena Carretón, Rodrigo Morchón

**Affiliations:** 1 Zoonotic Diseases and One Health Group, IBSAL-CIETUS (Biomedical Research Institute of Salamanca Research Centre for Tropical Diseases), Faculty of Pharmacy, University of Salamanca, 37007 Salamanca, Spain; 2 Department of Physiology and Pharmacology, Biomedical Research Institute of Salamanca (IBSAL), University of Salamanca, Salamanca, Spain; 3 Faculty of Veterinary Medicine, Research Institute of Biomedical and Health Sciences (IUIBS), University of Las Palmas de Gran Canaria, Arucas, 35413 Las Palmas, Spain

**Keywords:** Angiogenesis, Somatic antigens, *Dirofilaria repens* adult worms, Cell proliferation, Cell migration, Pseudo-capillary formation

## Abstract

**Background:**

Angiogenesis is defined as the formation of new vessels by sprouting of endothelial cells from pre-existing vessels in response to stimuli, such as hypoxia or inflammation. Subcutaneous dirofilariasis, caused by *Dirofilaria repens*, is a zoonotic disease characterized by the formation of subcutaneous nodules with the presence of at least one encapsulated worm, showing perivascular vascularization around it. The aim of this study is to analyze whether the somatic antigen of adult *D. repens* worms interacts with and modulates the angiogenic mechanism, cell proliferation and migration, and formation of pseudo-capillaries.

**Methods:**

The expression of VEGF-A, VEGFR-1/sFlt, VEGFR-2, mEnd and sEnd in cultures of human vascular endothelial cells stimulated with somatic antigen of adult worms of *D. repens* (DrSA), vascular endothelial growth factor (VEGF) and DrSA + VEGF were evaluated by using ELISA commercial kits. Cellular viability was analyzed by live cell count, cytotoxicity assays by using a commercial kit, cell proliferation by MTT-based assay, cell migration by wound-healing assay carried out by scratching wounds and capacity of formation of pseudo-capillaries analyzing cell connections and cell groups in Matrigel cell cultures. In all cases unstimulated cultures were used as controls.

**Results:**

DrSA + VEGF significantly increased the expression of VEGF-A, VEGFR-2 and mEndoglin compared to other groups and unstimulated cultures. Moreover, DrSA + VEGF produced cell proliferation and migration and increased the formation of pseudo-capillaries.

**Conclusions:**

Somatic antigen of adult *D. repens* worms activated the proangiogenic mechanism, cell proliferation and cell migration as well as formation of pseudo-capillaries in this in vitro human endothelial cell model. These processes could be related to the survival of adult *D. repens* in subcutaneous nodules in infected hosts.

**Graphical Abstract:**

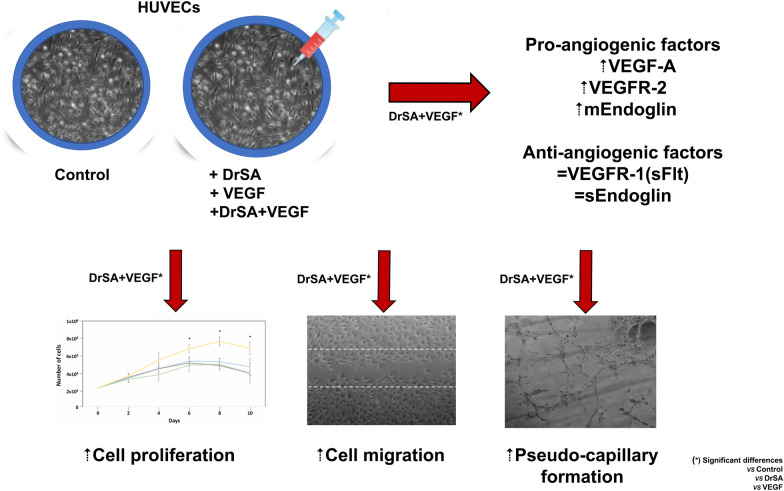

## Introduction

Angiogenesis is defined as the formation of new vessels by sprouting of endothelial cells from pre-existing vessels in response to stimuli such as hypoxia or inflammation [1–3]. A series of morphogenetic changes occur, consisting of endothelial cell activation, extracellular matrix degradation, endothelial cell proliferation and migration, vascular lumen formation, and vessel stabilization and maturation [4]. Endothelial cells produce a series of factors in response to these processes, including vascular endothelial growth factor (VEGF), which stimulates endothelial cells in adjacent vessels to grow and form new vessels [2, 5]. Increased levels of VEGF-A are detected by endothelial cells through binding to its tyrosine kinase-like receptor VEGFR-2, at which point a conformational change occurs that results in receptor dimerization and, via endothelial cells, triggers the release of nitric oxide and increased vascular permeability [5, 6]. However, both VEGFR-1 and its soluble form (sFlt1) exert negative regulation of signaling through VEGFR-2, as they act by sequestering the ligand and preventing it from binding to the receptor [7]. Endoglin is a vascular protein that plays a fundamental role in endothelial and vascular physiology, highlighting processes such as angiogenesis and vascular remodeling [8–10]. Endoglin expression increases in areas where vascular injury and active angiogenesis are taking place, in both tumor and non-tumor cells [11–15]. High concentrations of soluble endoglin (sEndoglin) have been described in patients with cancer, pre-eclampsia or cardiac conditions; in addition, antiangiogenic properties have been attributed to it, as it can prevent the correct development of angiogenesis in vivo and in vitro [16].

Subcutaneous dirofilariasis is a zoonotic disease, caused by *Dirofilaria repens*, which mainly affects canine reservoirs, both domestic and wild, and humans. In addition, it is a vector-borne disease that mainly affects Old World countries [17]. Like other filarial species, *D. repens* harbors intracellular symbiont bacteria of the genus *Wolbachia* whose contribution to inflammatory processes is key [18, 19]. Human subcutaneous dirofilariasis usually presents as a local inflammation at the subcutaneous level, which causes a nodule to form where the worm is encapsulated and destroyed [20]. In patients with subcutaneous nodules, ultrasound and Doppler techniques have shown that a clear peripheral vascularization develops around these nodules [21].

There are no studies analyzing the angiogenic character of *D. repens* but there are studies on other nematodes such as *Trichinella spiralis* and *Dirofilaria immitis*. In the first case, it has been shown that encapsulated larvae initiate angiogenesis and attract a set of highly permeable blood vessels to the surface of their collagenous capsule present in the musculature for nutrient acquisition and waste elimination, thus maintaining a long-term host-parasite relationship [22, 23]. Regarding *D. immitis*, Zueva et al. [24, 25] observed a proangiogenic effect of somatic antigens of *D. immitis* adults and an antiangiogenic effect of *Wolbachia* spp. In addition, in other diseases caused by lymphatic nematodes, it is suggested that microfilariae and adult filariae induce lymphangiogenesis and in vitro remodeling of lymphatic channels [26].

Against this background, the aim of our study was to determine whether *D. repens* is involved in the stimulation of the angiogenic process and in the cell proliferation and migration and the formation of pseudo-capillaries from adult worms located within subcutaneous nodules using an in vitro model of human endothelial cells.

## Methods

### Cell culture

Human umbilical vein endothelial cells (HUVECs) were grown in Endothelial Basal Medium 2 (Lonza, Walkersville, MD, USA) supplemented with SingleQuots^®^ (Lonza): 20% fetal bovine serum, heparin (22.5 µg/ml), VEGF (0.5 ng/ml), ascorbic acid (1 µg /ml), hFGF-B (10 ng/ml), hydrocortisone (0.2 µg /ml), hEGF (5 ng/ml), gentamicin (30 mg/ml), amphotericin B (15 µg/ml) and R^3^-IGF-3 (20 ng/ml). Plates were pre-coated with 0.1% pig gelatin (Sigma-Aldrich, Saint Louis, MO, USA), 0.01% fibronectin (Sigma-Aldrich) and 0.01% collagen (Corning). Cells were cultured at 37 °C in a humidified atmosphere in the presence of 5% CO_2_/95% air. The medium was changed every 3 days. Expansion was carried out by trypsinizing the cells (Trypsin/EDTA, Lonza) and replating them when the proliferating cells had reached a sufficient density. Passaging was performed at the ratio of 1:3. Cell counts were performed using a Countess® Automated Cell Counter (Invitrogen, Carlsbad, CA, USA) following the manufacturer’s instructions.

### Reagents and stimulation of endothelial cells, cytotoxity and cellular viability

Adult *D. repens* somatic antigens (DrSA) were prepared as previously described [27] and stored at −80 °C until use. In brief, *D. repens* adult worms (5) from a human skin nodule [28] was washed, macerated and sonicated in PBS, pH 7.2. The homogenate was centrifuged at 10,000*g*/30 min and the sediment discarded. The supernatant was the somatic antigenic extract employed for stimulations. Protein concentration was measured by DC protein assay commercial kit (Bio-Rad).

HUVECs were treated as previously described by Morchón et al. [27]. In brief, endothelial cells (10^6^ cells/plate) were plated on 60-mm culture plates and were grown for 4 days to obtain confluent cultures and treated with three different stimuli: 1 μg/ml of DrSA or Vascular Endothelial Growth Factor (VEGF) (R&D SYSTEMS) and 1 μg/ml of DrSA plus 1 μg/ml of VEGF (DrSA + VEGF). Unstimulated cells were used as controls in the same conditions. Stimulated and unstimulated cell cultures were carried out in triplicate. Finally, the supernatant of the cell cultures was collected, and HUVECs were lysed in ice-cold lysis buffer [20 mM Tris–HCl (pH 7.5); 140 mM NaCl; 10 mM ethylendiaminetetraacetic acid; 10% glycerol; 1% Igepal CA-630; aprotinin, pepstatin and leupeptin at 1 μg/ml each; 1 mM phenylmethylsulfonyl fluoride and 1 mM sodium orthovanadate].

Cytotoxicity was assessed in the supernatant of the stimulated and control cell cultures using the Toxilight BioAssay Kit (Cambrex, Verviers, Belgium) following the commercial instructions. This commercial kit quantitatively measures the release of adenylate kinase from damaged cells. Cellular viability was analyzed by cell counts using the Countess^®^ Automated Cell Counter (Invitrogen) following the manufacturer’s instructions. The results are presented as the mean ± SEM of three experiments performed in duplicate.

### Angiogenic factors assays

VEGF-A, VEGFR-1/sFlt, VEGFR-2 and sEndoglin concentrations in the endothelial cells culture medium were measured by ELISA using a Human VEGF-A Quantikine ELISA kit (R&D Systems, Minneapolis, MN, USA), Human VEGFR-1/sFlt Quantikine ELISA kit (R&D Systems), Human VEGFR-2 Quantikine ELISA kit (R&D Systems) and Human Endoglin Quantikine ELISA kit (R&D Systems), respectively, and membrane Endoglin (mEndoglin) concentration in the lysed endothelial cells was measured by Human Endoglin Quantikine ELISA kit (R&D Systems) following the manufacturers’ instructions. The results are presented as the mean ± SEM of three experiments performed in duplicate.

### Proliferation assays

Proliferation assays were assessed as previously described [29], with some modifications. In brief, 1000 cells were seeded on a 96-well plate and stimulated in complete HUVEC medium with 1 μg/ml DrSA, Vascular Endothelial Grown Factor (VEGF) (RRD SYSTEMS), 1 μg/ml DrSA plus 1 μg/ml VEGF and 1 μg/ml Cut plus 1 μg/ml of VEGF for 10 days. Unstimulated cells were used as controls in the same conditions. Proliferation at different days (every 2 days) of culture was determined by incubating cell cultures with 0.5 mg/ml 3-[4.5-dimethylthiazol-2-yl]-2.5-diphenyl tetrazolium bromide (MTT) (Sigma-Aldrich, St. Louis, MO, USA) for 4 h. Then, 10% SDS in 0.01 M HCl was added at a 1:1 (v/v) ratio and left overnight at 37 °C. Finally, absorbance was measured at 570 nm. The results are presented as the mean ± SEM of three experiments performed in triplicate.

### Migration assays

Wound-healing assays were assessed as previously described by González-Miguel et al. [30] with some modifications. In brief, *in vitro* scratched wounds were created by scraping confluent cell monolayers in 60-mm sterile plates with a sterile disposable pipette tip. The remaining cells were washed with sterile PBS buffer, incubated with the endothelial supplemented medium and stimulated with five different stimuli up to 6 h. Unstimulated cells were used as controls in the same conditions. Endothelial cell migration into the denuded area was monitored by photographing the plates every 30 min. The results are presented as the mean ± SEM of three experiments performed in duplicate.

### Endothelial cell tube formation assay

Endothelial cell tube formation was assessed as previously described by Jerkic et al. [31] with some modifications. In brief, a total of 8000 HUVECs per well were plated on Matrigel^®^ precoated µ-Slide Angiogenesis^®^ plates (Ibidi, Gräfelfing, Germany) in complete HUVEC medium with DrSA, VEGF and DrSA + VEGF (1:10 dilution). After seeding on Matrigel®, cells spread and aligned with each other to develop hollow tube-like structures. The cells and intercellular junctions were observed every 30 min for 5 h of incubation, and the morphological changes were photographed at 3 h using a phase contrast inverted Leica microscope (Leica, Wetzlar, Germany). Subsequently, the intercellular junctions were divided between the cell bodies to calculate the relationship between them (endothelial cell tube formation = cellular connections/cellular bodies). Unstimulated cells were used as controls in the same conditions. Each experiment was performed in triplicate.

### Statistical analysis

GraphPad Prism v.7 was used for all data analyses. Analyses were performed by ANOVA and corrected for repeated measurements when appropriate. If ANOVA revealed overall significant differences, individual means were evaluated post hoc using Tukey’s test. All results were expressed as the mean ± SEM. In all experiments, a significant difference was defined as a *p* value < 0.05.

## Results

### Effect of DrSA on cell viability, cytotoxicity and angiogenic factors

To determine whether *D. repens* is able to modify the production of some angiogenic factors, we analyzed the production of VEFG-A, VEGFR-1/sFLt, VEGFR-2, mEndoglin and sEndoglin in in vitro cultures of endothelial cells stimulated with DrSA, VEGF and DrSA + VEGF, where unstimulated cultures were used as controls.

No differences were found in cell viability and cytotoxicity of stimulated cultures with DrSA, VEGF and DrSA + VEGF compared to unstimulated cell cultures (data not shown).

**Fig. 1:**
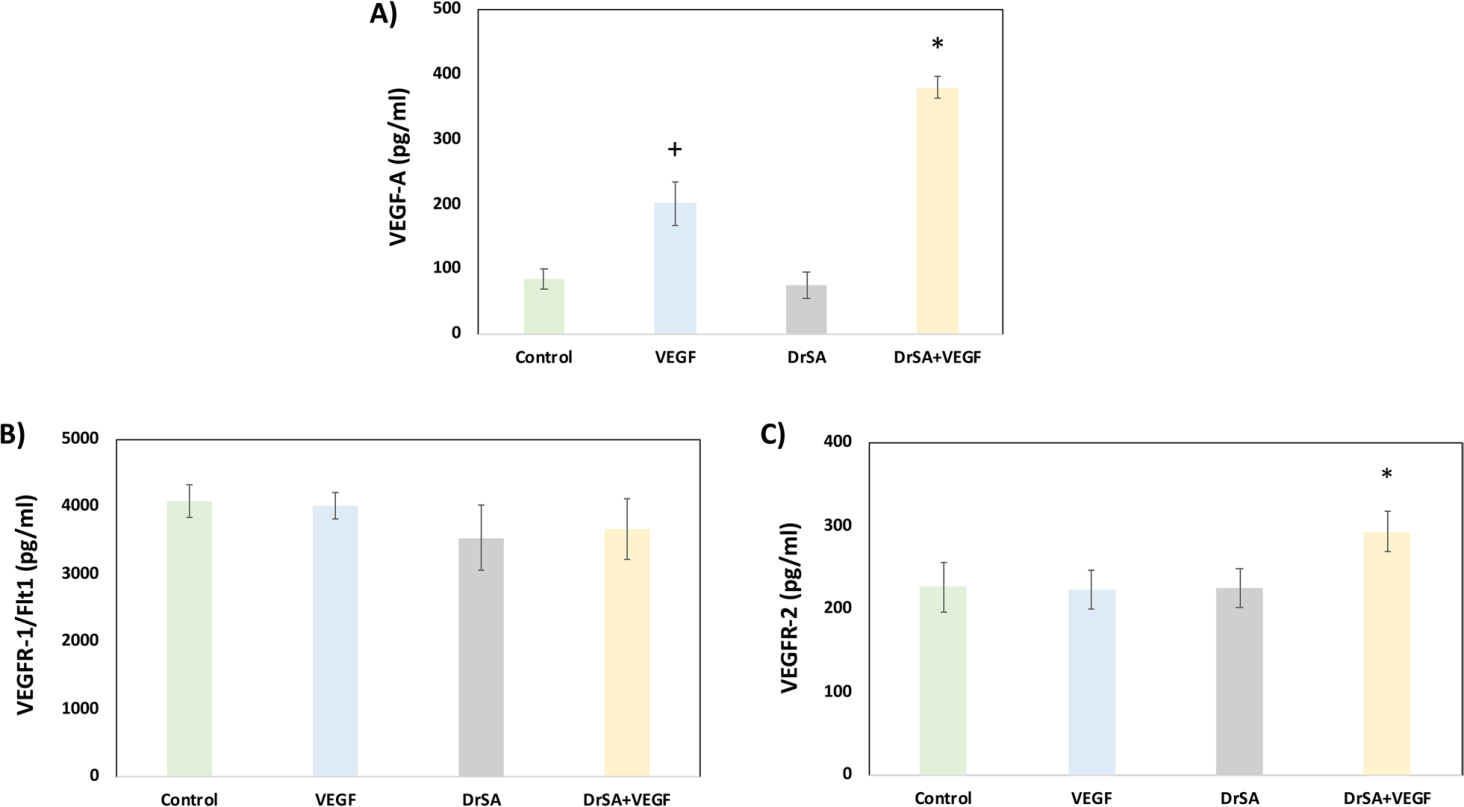
Effects of DrSA and Cut antigens on VEGF (**A**), VEGFR-1/sFlt1 (**B**) and VEGFR-2 (**C**) in unstimulated cultures () and cultures stimulated with VEGF (), DrSA () and DrSA + VEGF (). Results are expressed as the mean ± SEM of three independent experiments. The asterisk or plus sign (*/+) indicates significant differences (*p* < 0.05): DrSA + VEGF vs. control, VEGF and DrSA (*) and VEGF vs. control and DrSA (+)

The stimulation of cell cultures with DrSA + VEGF significantly increased the expression of VEGF-A compared to cell cultures stimulated with DrSA (*t* = 63.70, *df* = 4, *P* < 0.0001), VEGF (*t* = 40.28, *df* = 4, *P* < 0.0001) and unstimulated cultures (*t* = 63.82, *df* = 4, *P* < 0.0001). In addition, VEGF-stimulated cell cultures showed a significant increase in VEGF-A production compared to DrSA (*t* = 21.76, *df* = 4, *P* < 0.0001) and unstimulated cultures (*t* = 18.97, *df* = 4, *P* < 0.0001) (Fig. 1A). In addition, there were no significant differences for VEGFR-1/sFlt between stimulated and unstimulated cell cultures (Fig. 1B), and only VEGFR-2 was detected in DrSA + VEGF stimulated cell cultures. In brief, DrSA + VEGF stimulated cell cultures showed a significant increase compared with DrSA (*t* = 8.802, *df* = 2, *P* = 0.0127), VEGF stimulated cultures (*t* = 5.364, *df* = 2, *P* = 0.033) and unstimulated cultures (*t* = 6.484, *df* = 2, *P* = 0.023) (Fig. 1C).

**Fig. 2:**
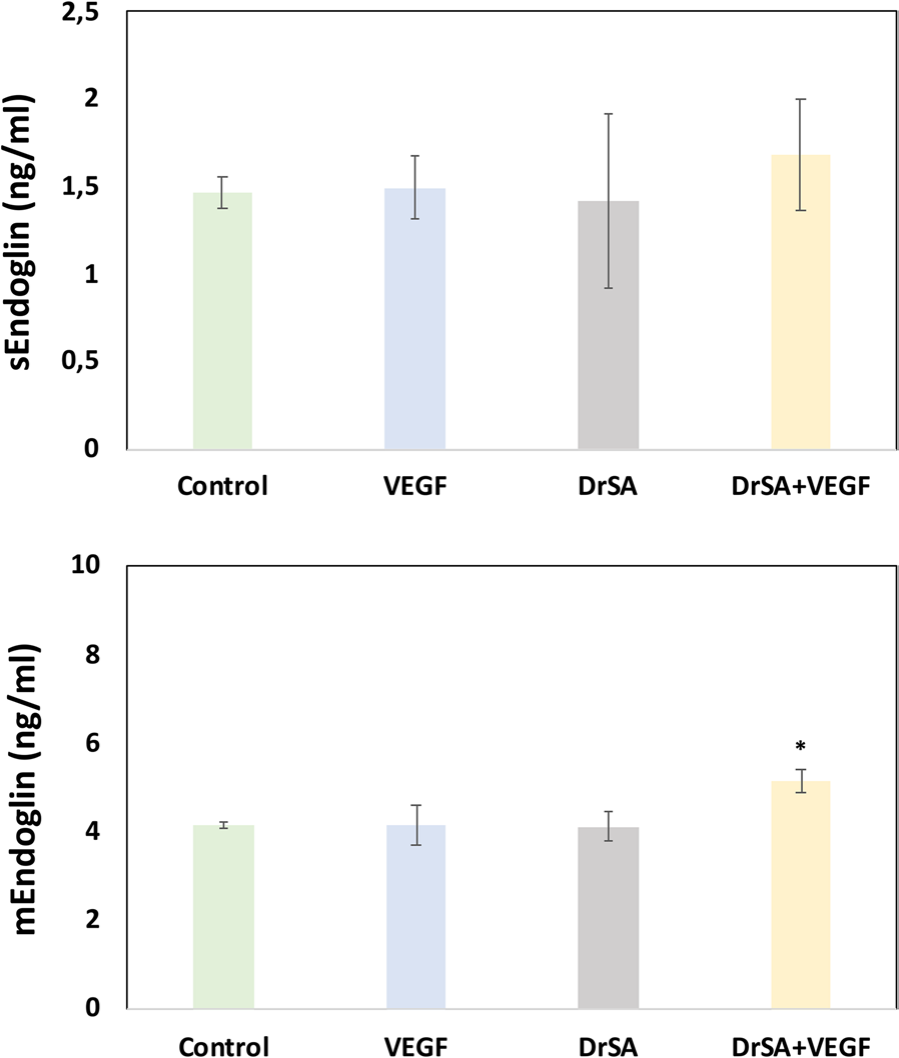
Effects of DrSA and Cut antigens on sEndoglin and mEndoglin in unstimulated cultures () and cultures stimulated with VEGF (), DrSA () and DrSA + VEGF (). Results are expressed as the mean ± SEM of three independent experiments. Significant differences (*) in comparisons with the other groups are indicated (*p* < 0.05)

The stimulation of cell cultures with DrSA + VEGF only significantly increased the expression of mEndoglin when compared to cell cultures stimulated with DrSA (*t* = 6.46, *df* = 2, *P* = 0.0231), VEGF (*t* = 4.559, *df* = 2, *P* = 0.0449) and unstimulated cultures (*t* = 5.112, *df* = 2, *P* = 0.0362). However, when we analyzed the expression of sEndoglin, no significant differences were observed between stimulated and unstimulated cultures (Fig. 2).

### DrSA produces cell proliferation

**Fig. 3:**
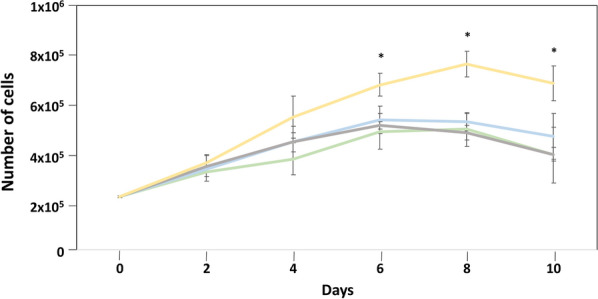
Effects of DrSA and Cut antigens on cell proliferation in unstimulated cultures () and cultures stimulated with VEGF (), DrSA () and DrSA + VEGF (). Results are expressed as the mean ± SEM of three independent experiments. Significant differences (*) in comparisons with the other groups are indicated (*p* < 0.05)

The effect of DrSA on the proliferation of endothelial cells was quantified using the MTT technique in a 10-day period (Fig. 3). All cultures showed typical cell growth curves in all experimental groups with a progressive growth between days 0 and 6 or 8 post-stimulation, experiencing a decrease of viable cells from there until day 10 post-stimulation. MTT technique showed a significant increase in the number of viable cells on day 6 post-stimulation in cultures stimulated with DrSA + VEGF compared with cultures stimulated with DrSA (*t* = 5.346, *df* = 4, *P* = 0.0059), VEGF (*t* = 3.139, *df* = 4, *P* = 0.0349) and unstimulated cultures (*t* = 3.45, *df* = 4, *P* = 0.0251) on day 8 post-stimulation in cultures stimulated with DrSA + VEGF compared with cultures stimulated with DrSA (*t* = 7.051, *df* = 4, *P* = 0.0021), VEGF (*t* = 5.68, *df* = 4, *P* = 0.0047) and unstimulated cultures (*t* = 4.711, *df* = 4, *P* = 0.0092) and on day 10 post-stimulation in cultures stimulated with DrSA + VEGF compared with cultures stimulated with DrSA (*t* = 5.914, *df* = 4, *P* = 0.0041), VEGF (*t* = 2.878, *df* = 4, *P* = 0.0451) and unstimulated cultures (*t* = 3.424, *df* = 4, *P* = 0.0267).

### DrSA produces cell migration

**Fig. 4:**
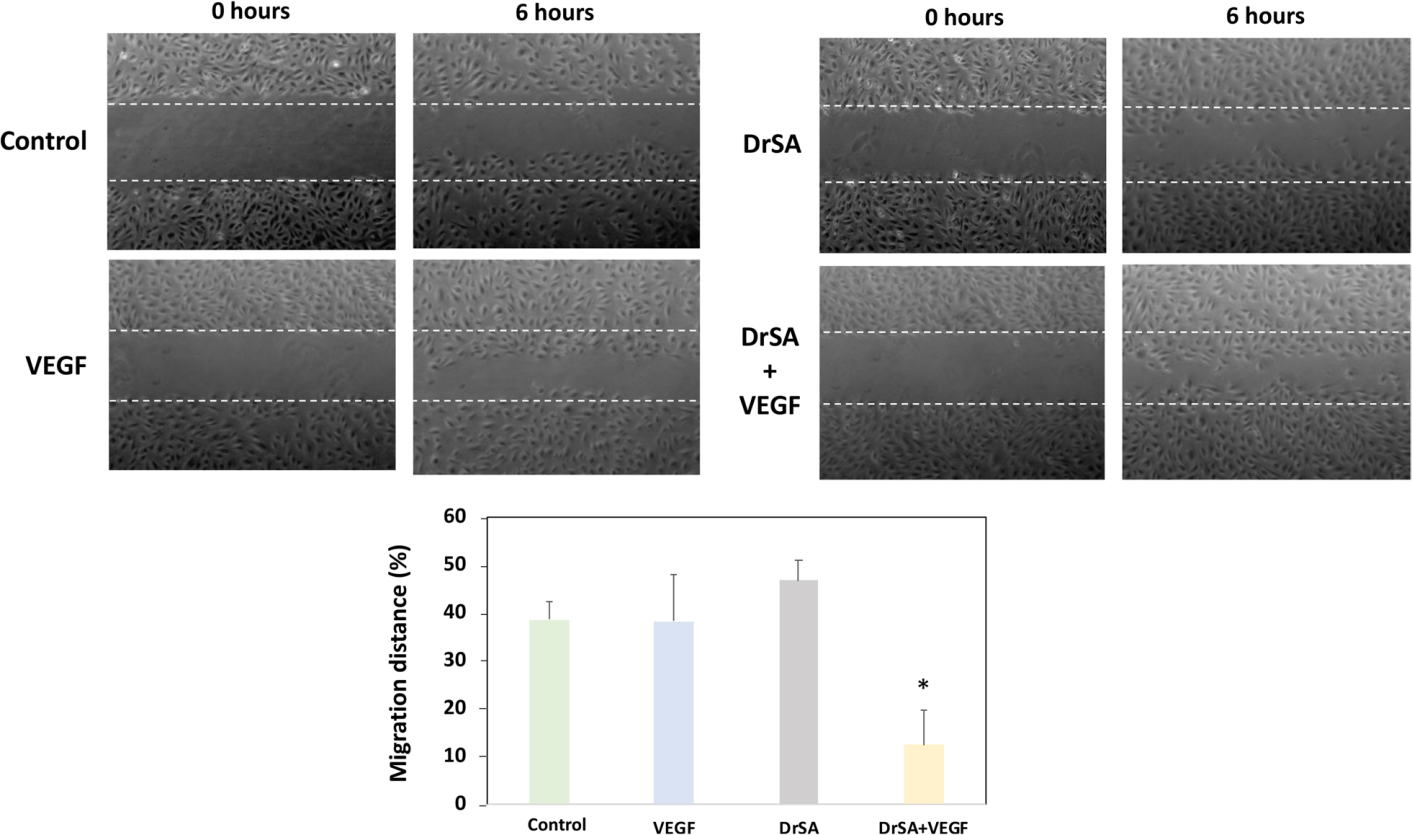
Effects of DrSA and Cut antigens on cell migration distance in unstimulated cultures () and cultures stimulated with VEGF (), DrSA () and DrSA + VEGF (). Results are expressed as the mean ± SEM of three independent experiments. Significant differences (*) compared with the other groups are indicated (*p* < 0.05)

A wound-healing assay was performed to assess migration of endothelial cells (Fig. 4). The quantification was carried out by measuring the distance of migration compared with negative control (untreated cells) to 6 h post-stimulation. A significant decrease of distance migration after stimulation with DrSA + VEGF with respect to DrSA (*t* = 12.5, *df* = 2, *P* = 0.002) and VEGF (*t* = 4.853, *df* = 2, *P* = 0.0083) stimulated and unstimulated cultures (*t* = 10.84, *df* = 2, *P* = 0.0004).

### Effect of DrSA on pseudo-capillary formation

**Fig. 5:**
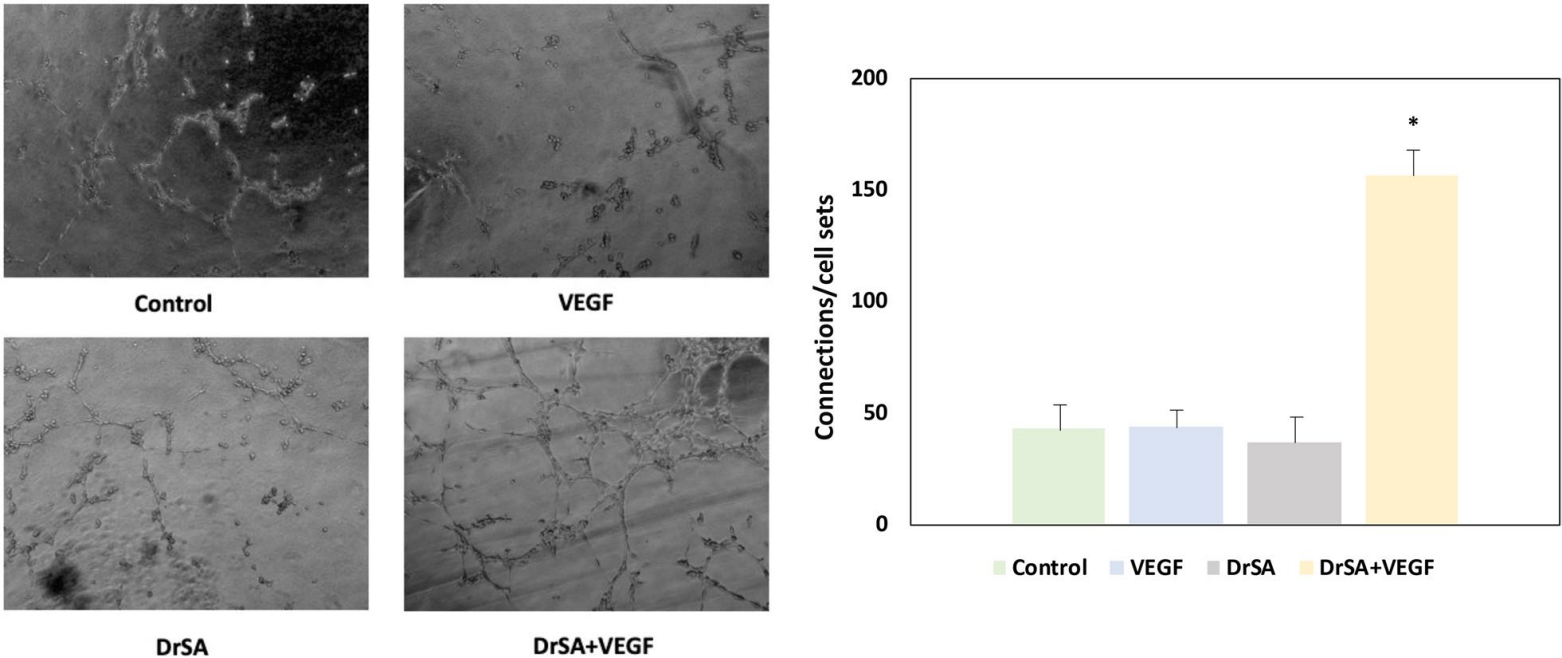
Effects of DrSA and Cut antigens on connections and cellular set in unstimulated cultures () and cultures stimulated with VEGF (), DrSA () and DrSA + VEGF (). Results are expressed as the mean ± SEM of three independent experiments. Significant differences (*) compared with the other groups are indicated (*p* < 0.05)

The capacity for pseudo-capillary formation was evaluated by analyzing the cell junctions (connections) and the cellular set that emerged in stimulated and unstimulated cell cultures (Fig. 5). The formation of pseudo-capillaries and the connections/joint relationship in cultures stimulated with DrSA + VEGF showed a significant increase compared to cell cultures stimulated with DrSA (*t* = 7.74, *df* = 2, *P* = 0.0163), VEGF (*t* = 7.159, *df* = 2, *P* = 0.019) and unstimulated cultures (*t* = 6.514, *df* = 2, *P* = 0.0228).

## Discussion

Subcutaneous dirofilariasis (*D. repens*) is a vector-borne zoonotic disease mainly affecting canids and humans, which causes the formation of subcutaneous nodules in most cases [32]. *Dirofilaria repens* has been shown to be able to develop mechanisms that allow it to lengthen the survival of the parasite in the host, including the formation of subcutaneous nodules and modulation of the immune response, among others [19].

There are no studies analyzing the angiogenic character of *D. repens* but there are studies on other nematodes such as *T. spiralis*, in which larvae initiate angiogenesis and attract a set of highly permeable blood vessels to the surface of the collagenous capsule present in the musculature to achieve nutrient acquisition, waste elimination and thus maintain a long-term host-parasite relationship [22, 23]. The role of *D. immitis* and *Wolbachia* in the angiogenic process has also been studied. In fact, the somatic antigen of *D. immitis* promotes the production of angiogenic molecules, while *Wolbachia* and adult *D. immitis* worms from dogs treated with doxycycline are able to stimulate anti-angiogenic molecules and decrease pseudo-capillary formation [24, 25]. In other lymphoid nematodes, it is suggested that microfilariae and adult filariae induce lymphangiogenesis and in vitro remodeling of lymphatic channels, which would demonstrate that the parasites stimulate mechanisms to promote vascular supply in damaged tissues [26]. In patients with subcutaneous nodules caused by *D. repens*, ultrasound and Doppler techniques have shown that peripheral vascularization is evident around these nodules [21].

The aim of this study was to determine whether adult *D. repens* worms could stimulate the angiogenic process (formation of new blood vessels from pre-existing vessels) at the endothelial level. To recreate the conditions under which the angiogenic process is initiated by endothelial cells after an obstructive or hypoxic process, among others, human endothelial cells were stimulated with VEFG, the first factor that occurs in the angiogenic process [33], and DrSA.

First, neither DrSA nor VEGF produced a cytotoxic effect or altered endothelial cell viability. Second, DrSA + VEGF significantly stimulated VEGF-A and VEGFR-2 production compared to VEGF-produced stimulations and in unstimulated cells. Both molecules are potent proangiogenic mediators that have mitogenic and anti-apoptotic effects on endothelial cells and are able to inhibit the host immune response, among other functions [26, 34]. A similar effect occurred in macrophage and mast cell culture stimulated with antigens of encapsulated larvae of *T. spiralis* [35, 36] and in endothelial cells stimulated with somatic antigen of adult *D. immitis* [18, 24, 27]. In addition, some authors suggested that VEGF is a key factor for the formation of new vessels around nurse cells in parasitic nematodes [37]. However, the levels of VEGFR-1/sFlt-1 were not modified, similar to studies carried out by Zueva et al. [24, 25], where their production was analyzed in canine endothelial cells stimulated with somatic extracts of *D. immitis* derived from dogs untreated and treated with doxycycline (with lesser amounts of *Wolbachia*) and recombinant *Wolbachia* Surface Protein. These results may indicate that VEGFR-1/sFlt-1 does not participate in the angiogenic process for at least the first 24 h.

Third, only DrSA + VEGF increased mEndoglin expression without altering sEndoglin expression compared the other stimulated and unstimulated cultures. mEndoglin is the cell membrane-bound form of endoglin, which causes a proangiogenic effect, and its expression has been observed to increase under physiological conditions during tissue vascularization as well as in pathological conditions including angiogenesis [24]. In other studies, mEndoglin production decreased when endothelial cell cultures were stimulated by Wolbachia [25], while sEndoglin production (related to anti-angiogenic processes [3]) increased. Although adult *D. repens* worms contain the endosymbiont *Wolbachia* bacteria [38], *Wolbachia* does not appear to be a determinant when *D. repens* proteins are in the majority, as in the case of *D. immitis* [24], but is a determinant when it is in the majority [25] or when it has previously been eliminated [24] and anti-angiogenic mechanisms are stimulated.

Fourth, the present study analyzed whether cell proliferation and migration processes were affected when stimulation with DrSA and VEGF was performed in our HUVEC model, and the results showed that both processes were affected, with increased cell proliferation and migration observed in endothelial cell cultures stimulated with DrSA + VEGF and unaffected by the other stimuli. VEGF production seemed able to promote cell proliferation and migration and to inhibit the host immune response [3, 26, 31, 39], which are closely related to vasculogenesis and angiogenesis. In studies carried out in other canine endothelial cell models, *D. immitis* seemed to increase cell proliferation and migration within the fibrinolytic process, which is related to angiogenesis [30]. Therefore, these results confirmed previous findings that the proangiogenic process was favored when the endothelial cell culture was stimulated with DrSA + VEGF.

Finally, the effect of DrSA on the formation of vascular pseudo-capillaries was analyzed. These structures form on a Matrigel matrix [40], which simulates the formation of immature vessels that form during angiogenesis. In the present human endothelial cell model, only DrSA + VEGF produced a significant increase in the formation of pseudo-capillaries, which is similar to previous results. However, other studies have shown that the presence of *Wolbachia* significantly decreased pseudo-capillary formation in canine endothelial cells [25], which is related to anti-angiogenic processes. In this study, the effect of somatic antigen from adult *D. repens* worms, which contains *Wolbachia* [38], has been shown to be contrary to this fact, so that the amount of *Wolbachia* used alone or as a minority part of the protein load of adult *D. repens* worms in the host may condition the drift of the angiogenic process.

## Conclusions

The results obtained in the present study provide the first data on the angiogenic effect produced by adult *D. repens* worms together with VEGF in human endothelial cells. This effect favors the production of proangiogenic molecules, cell proliferation and migration as well as the formation of pseudo-capillaries, which could facilitate parasite survival by favoring the formation of new vessels surrounding subcutaneous nodules. Further studies are needed to investigate the effect of these antigens on the angiogenic process and on other mechanisms in which a direct parasite-host interaction is established, aiming to facilitate the survival of the parasite in the hosts.

## Data Availability

The datasets supporting the conclusions of this article are included within the article.

## Abbreviations

VEGF: Recombinant human vascular endothelial growth factor
VEGF-A: Vascular endothelial grown factor A
VEGFR-1/sFlt: Soluble vascular endothelial growth factor receptor 1
VEGFR-2: Vascular endothelial growth factor receptor 2
mEndoglin: Membrane endoglin
sEndoglin: Soluble endogli
DrSA: Adult *D. repens* somatic antigens
DC: Detergent compatible
*t*: Tukey value
*df*: The number of degrees of freedom
